# Screening for obstructive sleep apnoea in post‐treatment cancer patients

**DOI:** 10.1002/cnr2.1740

**Published:** 2022-12-13

**Authors:** Harini Subramanian, Veronika Fuchsova, Elisabeth Elder, Alison Brand, Julie Howle, Anna DeFazio, Graham J. Mann, Terence Amis, Kristina Kairaitis

**Affiliations:** ^1^ Ludwig Engel Centre for Respiratory Research The Westmead Institute for Medical Research Westmead Australia; ^2^ Westmead Clinical School, Faculty of Medicine and Health The University of Sydney Westmead Australia; ^3^ Breast Cancer Institute Westmead Hospital Westmead Australia; ^4^ Department of Gynaecological Oncology Westmead Hospital Westmead Australia; ^5^ Crown Princess Mary Cancer Centre Westmead and Blacktown Hospitals Blacktown Australia; ^6^ Melanoma Institute Australia The University of Sydney Camperdown Australia; ^7^ Centre for Cancer Research The Westmead Institute for Medical Research Westmead Australia; ^8^ The Daffodil Centre The University of Sydney Camperdown Australia; ^9^ John Curtin School of Medical Research Australian National University Canberra Australia; ^10^ Department of Respiratory and Sleep Medicine Westmead Hospital Westmead Australia

**Keywords:** breast cancer, cancer outcomes, endometrial cancer, melanoma, screening, sleep apnea

## Abstract

**Background and aims:**

For cancer patients, comorbid obstructive sleep apnea (OSA) poses additional risk to their surgical/anaesthetic outcomes, quality of life, and survival. However, OSA screening is not well‐established in oncology settings. We tested two screening tools (STOP‐Bang questionnaire [SBQ] and the at‐home monitoring device, ApneaLink™*Air*), for predicting polysomnography (PSG) confirmed OSA in post‐treatment cancer patients.

**Methods:**

Breast (*n* = 56), endometrial (*n* = 37) and melanoma patients (*n* = 50) were recruited from follow‐up clinics at Westmead Hospital (Sydney, Australia). All underwent overnight PSG, 137 completed SBQ, and 99 completed ApneaLink™*Air*. Positive (PPV) and negative (NPV) predictive values for PSG‐determined moderate‐to‐severe OSA and severe OSA, were calculated using an SBQ threshold ≥3 au and ApneaLink™*Air* apnoea‐hypopnea index thresholds of ≥10, ≥15 and ≥30 events/h.

**Results:**

Both SBQ and ApneaLink™*Air* had high NPVs (92.7% and 85.2%–95.6% respectively) for severe OSA, but NPVs were lower for moderate‐to‐severe OSA (69.1% and 59.1%–75.5%, respectively). PPV for both tools were relatively low (all <73%). Combining both tools did not improve screening performance.

**Conclusions:**

These screening tools may help identify cancer patients without severe OSA, but both are limited in identifying those with moderate‐to‐severe or severe OSA. PSG remains optimal for adequately identifying and managing comorbid OSA in cancer patients.

## 1 INTRODUCTION

Obstructive sleep apnea (OSA) has a reported prevalence of 9%–38% for adults.^1^ It is characterised by recurrent episodes of complete (apnoea) or partial (hypopnoea) upper airway collapse during sleep^2^ resulting in repetitive nocturnal oxygen desaturation events of varying severity and frequency.^2^ OSA has numerous recognised health impacts including; (1) reduced cognitive function and higher accident risk linked to daytime somnolence,^3^, ^4^ (2) poorer wellbeing and quality of life associated with OSA symptomology,^5^ (3) increased risk for longer‐term cardiovascular consequences, e.g. hypertension,^6^ (4) increased risk for stroke^7^ and (5) worsened post‐surgical outcomes.^8^ For cancer patients, co‐morbid OSA may pose additional risks, since epidemiological studies suggest associations with both higher cancer incidence^9^ and increased mortality rates.^10^

A number of published studies report relatively high prevalence rates for moderate‐to‐severe OSA (AHI≥15 events/h) in cancer patient cohorts such as; breast cancer (13.3%–58%),^11^, ^12^ endometrial cancer (57%),^11^ melanoma (60.7%),^13^, ^14^ lung cancer (50%)^15^, ^16^ and prostate cancer (48.7%).^17^ Overall, these prevalence rates tend to be higher than those reported (21.2%–49.7%) for community‐based cohorts,^18^, ^19^ and even approach those seen in sleep‐clinic settings (51.7%–67.4%).^20^, ^21^, ^22^, ^23^ Notably, OSA prevalence in breast and endometrial cancer patients,^11^ exceed rates observed in women from the general population (13.2%–23.4%)^18^, ^19^ and from women in sleep clinics (38.8%).^20^ These findings highlight the extent of unrecognised OSA likely present in cancer patient cohorts, making OSA a relatively prevalent co‐morbidity for cancer patients.

The aetiology of OSA in cancer cohorts is not well understood. However, a likely candidate is the presence of common risk factors. Among these, age and obesity are well‐known risk factors for both OSA^24^, ^25^, ^26^ and a number of cancers.^27^, ^28^, ^29^ OSA is a treatable condition, but is often left unrecognised, undiagnosed, and untreated.^30^, ^31^ Implementing protocols expediting OSA diagnoses in oncology‐clinic settings can facilitate timely OSA treatment, to reduce the burden of comorbid OSA in cancer patients.

An OSA diagnosis is made using overnight, attended, in‐laboratory polysomnography (PSG), or unattended home PSG, both of which are finite, labour intensive resources.^32^ Alternatively, tools such as questionnaires^33^, ^34^ and at‐home limited channel monitoring studies, which record between 2 and 8 physiological variables,^35^ can be used to identify patients with a high probability of OSA for streamlining the utilisation of finite PSG resources. This approach has been utilised in primary care,^36^ and pre‐anaesthetic clinics,^33^ but has not been widely applied in oncology clinics. The aim of the present study was to investigate the relative ability of two widely used and previously validated community/sleep clinic screening tools to accurately identify cancer patients with a high probability for OSA when deployed in an oncology clinic setting: (1) the STOP‐Bang questionnaire (SBQ),^33^ and (2) the ApneaLink™*Air* device (ResMed, Australia).^35^

We recruited patients from three different post‐treatment oncology clinics: (1) breast cancer; a female cohort with a reported high prevalence of sleep disturbance,^37^ (2) endometrial cancer; a female cohort with a high prevalence of obesity (a shared risk factor for OSA),^38^ and (3) melanoma; a mixed sex cohort with a proposed cancer‐OSA pathological linkage.^39^

## 2 MATERIALS AND METHODS

### 2.1 Patients

The Western Sydney Local Health District Ethics Committee approved this study. Written informed consent was obtained from all patients. The study was conducted in accordance with the Declaration of Helsinki.

Patients were recruited from melanoma, breast, or endometrial cancer outpatient clinics at Westmead Hospital, Sydney Australia; from August 2016 until November 2019. PSG data for breast and endometrial cancer patients included in the present study have been reported previously.^11^ Patients were eligible to participate if they: (1) were ≥ 18 years of age, (2) had a confirmed diagnosis of either breast cancer, endometrial cancer or melanoma, (3) had completed their treatment regimen (i.e., surgery, chemotherapy, radiotherapy) for a minimum of 2 months (endometrial cancer), 6 months (melanoma), or 12 months (breast cancer) prior to study recruitment, (4) were able to understand instructions relevant to study requirements, and (5) provided informed written consent. Patients were also screened for serious medical conditions via a physician‐conducted telephone interview, and were excluded if they had serious respiratory, cardiovascular, hepatic, renal, neurological or psychiatric conditions, or if they were pregnant.

### 2.2 Polysomnography

All patients underwent a standard clinical practice (single‐night)^32^ overnight in‐laboratory PSG session in the Sleep Research Facility, at the Westmead Institute for Medical Research (Sydney, Australia). The following signals were measured: nasal pressure, oronasal thermistor signals, snoring auditory signals, thoracic and abdominal inductive plethysmography, pulse oximetry (SpO_2_), EEG, EOG, and chin, diaphragm, and pre‐tibial EMG.

### 2.3 STOP‐Bang questionnaire

The SBQ is an eight‐item binary (yes/no) validated tool, measuring recognised OSA phenotypic characteristics.^33^ The ‘STOP’ section contains four questions, each capturing a self‐reported OSA symptom (i.e., snoring, tiredness, observed apnoea and high blood pressure). The ‘Bang’ section records four additional demographic conditions (BMI ≥35 kg/m^2^, age ≥ 50 years, neck circumference ≥40 cm and male gender). Patients self‐completed the questionnaire, prior to undergoing PSG.

### 2.4 ApneaLink™Air

Patients received an at‐home limited channel monitoring device kit (ApneaLink™*Air* kit; ResMed, Sydney, Australia) with printed instructions, either during recruitment or upon PSG completion. The device records respiratory airflow from a nasal cannula, respiratory effort via a thoracic movement ‘effort’ sensor, and blood oxygen saturation and pulse rate using a finger pulse oximeter. After self‐administered usage at home for one night, the kit was returned in‐person or via a pre‐paid postal envelope.

### 2.5 Data analysis

#### 2.5.1 Polysomnography

PSG data were collected, scored, and analysed by an experienced sleep technician using American Academy of Sleep Medicine (AASM‐2012) criteria.^39^ The following metrics were calculated: (1) total sleep time (TST_PSG_), (2) apnoea‐hypopnoea index (AHI_PSG_), (3) oxygen desaturation index (ODI_PSG_; number of desaturations of ≥3% per hour), and (4) proportion of time asleep when SpO_2_ levels were <90% (Tsat_PSG_ < 90%).

#### 2.5.2 STOP‐Bang questionnaire

‘Yes’ responses were scored ‘1,’ and ‘No’ responses were scored ‘0.’ A total SBQ score was determined by summing the ‘yes’ responses. The total SBQ score can range between 0 and 8 arbitrary units (au).^33^

#### 2.5.3 ApneaLink™Air data

ApneaLink™*Air* data were downloaded onto a computer for analysis. Data were analysed and scored automatically using commercially available software (ApneaLink™*Air* Application Software Multilingual, ResMed Australia). The evaluation period for each recording was the total recording period minus the first 10 min, and sections with poor quality signals. If the evaluation period was <120 min, the recording was excluded. The recordings were reviewed by an experienced sleep technician for acceptable technical quality and to confirm all identified events met AASM‐2012 criteria.^40^ Events were re‐scored by the sleep technician if required.

An event was scored as an apnoea if it involved a ≥ 90% drop in respiratory airflow from pre‐event baseline, lasting ≥10 s. Hypopneas were scored if events involved a ≥ 30% drop in respiratory airflow lasting ≥10 s. The following outputs were generated: (1) evaluation period (min), (2) apnoea‐hypopnea index (AHI_AL_: includes obstructive, central, and mixed apnoea classes and hypopneas per hour), (3) oxygen desaturation index (ODI_AL_: number of desaturations of ≥3% per hour) and (4) percentage of time asleep with SpO_2AL_ ≤ 90% (Tsat_AL_ ≤ 90%). Sleep duration was considered equivalent to the evaluation period.

#### 2.5.4 Statistical analysis

Statistical analysis was performed using Statistical Package for Social Sciences version 24 (IBM SPSS Inc., Chicago, Illinois, USA), GraphPad PRISM® version 9.3.1 (GraphPad Inc., San Diego, CA) and Microsoft Excel version 7 (Microsoft, Redmond, Washington, USA). Individual data were grouped and expressed as median (interquartile range [IQR]). Kruskal‐Wallis tests were used to compare continuous data, and chi‐square tests for categorical data. A two‐tailed *p‐*value <.05 was considered statistically significant.

The ability of the SBQ and ApneaLink™*Air* data to predict PSG‐confirmed OSA, was determined using positive predictive values (PPV) and negative predictive values (NPV).

1. $\mathrm{PPV}=\left(\text{True positive cases}/\text{Test positive cases}\right)\times 100.$
2. $\mathrm{NPV}=\left(\text{True negative cases}/\text{Test negative cases}\right)\times 100.$

For this study, an AHI_PSG_ ≥ 15 events/h was used to confirm the presence of moderate‐to‐severe OSA, while an AHI_PSG_ ≥ 30 events/h established severe OSA. PPV and NPV values were calculated for: (1) a SBQ ≥3 au, a threshold score previously established to possess high discriminative power to screen for moderate‐to‐severe and severe OSA,^33^, ^41^ (2) AHI_AL_ thresholds of 10, 15 and 30 events/h, and (3) a combination of SBQ≥3 au and the AHI_AL_ thresholds. PPV and NPV values for both SBQ and ApneaLink™*Air* data were also assessed by cancer type and age (<60 years vs. ≥60 years).

## 3 RESULTS

We recruited 56 breast cancer, 37 endometrial cancer and 50 melanoma patients (Table 1).

**TABLE 1:** Anthropometric and demographic characteristics of study cohort

	BRC (*n* = 56)	ENDO (*n* = 37)	MEL (*n* = 50)	Total (*n* = 143)	*p* ^a^
Age (years)	60.0 (54.0–67.0)	59.0 (54.5–67.0)	61.5 (52.8–69.0)	60.0 (54.0–67.0)	.8745
Gender (F/M)	56/0	37/0	18/32	111/32	<.0001
Height (cm)	160.0 (155.1–164.0)	160.0 (153.8–162.5)	171.1 (167.0–175.1)	162.0 (156.6–170.0)	<.0001
Weight (kg)	73.5 (65.7–83.8)	78.4 (72.0–101.1)	87.9 (76.6–95.1)	79.2 (69.2–93.3)	.0015
BMI (kg/m^2^)	29.1 (25.6–31.3)	32.6 (28.4–38.0)	30.0 (26.0–33.0)	29.9 (26.4–33.5)	.0035
Neck circumference (cm)	35.0 (33.6–37.0)	37.0 (34.5–39.5)	40.0 (35.5–42.9)	36.5 (34.0–40.5)	.0004

*Note*: Data are median (IQR).

Abbreviations: BMI, body‐mass index, BRC, breast cancer, ENDO, endometrial cancer; *F*, female; M, male; MEL, melanoma; *n*, number of patients.

^a^ *p*‐value obtained comparing cancer subgroups via Kruskal‐Wallis or chi‐squared tests, as appropriate.

### 3.1 Patient characteristics

Anthropometric and demographic data for all patients are displayed in Table 1. PSG data were obtained from 143 patients, with SBQ data available for 137 of these patients. The ApneaLink™*Air* device was provided to 117 patients, however only 99 patients had recordings meeting technical criteria (see 2.5.3). There were 53 patients with PSG data who had both an SBQ score ≥3 au and a technically acceptable ApneaLink™*Air* recording.

### 3.2 Polysomnography

PSG metrics and prevalence values for moderate‐to‐severe and severe OSA, stratified by cancer subgroup, are displayed in Table 2.

**TABLE 2:** Polysomnography metrics and prevalence of moderate‐to‐severe and severe OSA within the study cohort, stratified by cancer subgroup

	BRC (*n* = 56)	ENDO (*n* = 37)	MEL (*n* = 50)	Total (*n* = 143)	*p* ^a^
PSG metrics					
TST_PSG_ (min)	357.5 (313.3–416.8)	372.0 (313.8–419.3)	353.0 (324.4–386.1)	356.5 (318.0–398.0)	.4125
AHI_PSG_ (events/h)	17.5 (7.4–34.8)	15.7 (10.0–33.4)	12.8 (7.4–22.0)	14.6 (7.5–31.6)	.2421
ODI_PSG_ (events/h)	5.7 (2.1–17.0)	10.0 (2.4–20.9)	3.3 (1.9–8.8)	5.5 (2.1–15.1)	.0613
Tsat_PSG_ < 90% (min)	0.4 (0.0–3.5)	2.4 (0.2–9.7)	0.2 (0.0–1.1)	0.5 (0.0–3.3)	.0014
OSA severity categories					
AHI_PSG_ ≥ 15 (*n* [%])	30 (53.6%)	21 (56.8%)	20 (40.0%)	71 (49.7%)	.2283
AHI_PSG_ ≥ 30 (*n* [%])	17 (30.4%)	12 (32.4%)	8 (16.0%)	37 (25.9%)	.1382

*Note*: Data are median (IQR) or *n* (%) respectively.

Abbreviations: AHI, apnoea‐hypopnea index (events/h); AHI_PSG_, AHI obtained via polysomnography; AHI_PSG_ ≥ 15 events/h, moderate‐to‐severe OSA; AHI_PSG_ ≥ 30 events/h, severe OSA; BRC, breast cancer; *n*, number of patients; %, % of respective group; ENDO, endometrial cancer; MEL, melanoma; ODI_PSG_, ODI obtained via polysomnography; PSG, polysomnography; TST_PSG_, total sleep time obtained via polysomnography; Tsat_PSG_ < 90%, time when blood oxygen saturation < 90% obtained via polysomnography.

^a^ *p*‐value obtained comparing cancer subgroups via Kruskal‐Wallis or chi‐squared tests, as appropriate.

### 3.3 SBQ scores

**FIGURE 1:**
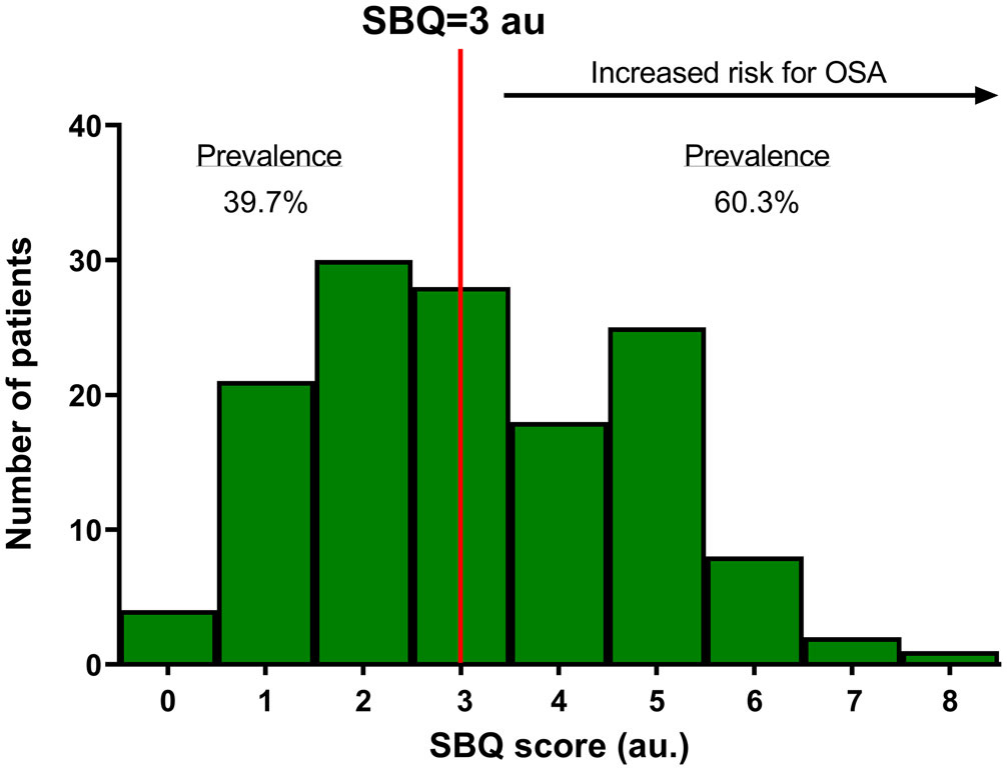
Bar chart showing distribution of STOP‐Bang questionnaire (SBQ) scores (*n* = 137)

The median (IQR) SBQ score was 3 (2.0–5.0) au., with 82 patients scoring ≥3 au (Figure 1).

### 3.4 ApneaLink™Air data

Of the 117 patients who received an ApneaLink™*Air* device, 12 (10.3%) did not subsequently use the device. Recordings from 6 (5.7%) of the 105 patients who used the device, were considered technically unacceptable. Overall, the failure rate for obtaining technically acceptable recordings from those who received an ApneaLink*™Air* was 15.4%.

**FIGURE 2:**
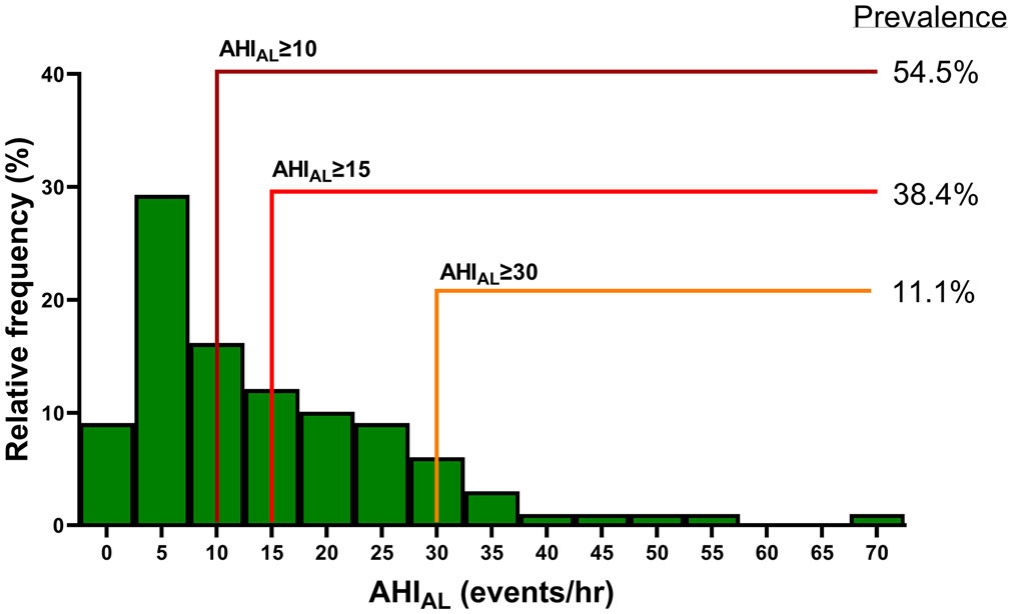
Frequency histogram for ApneaLink™*Air*‐derived apnoea‐hypopnea indices (AHI_AL_) values (*n* = 99). Bin width = 5 events/h. Coloured lines represent indicated AHI_AL_ threshold values, with associated percentages indicating prevalence for each AHI_AL_‐determined OSA severity category.

The prevalence of ApneaLink*™Air*‐determined moderate‐to‐severe OSA (*AHI*_*AL*_ ≥ 15 events/h) and severe OSA (*AHI*_*AL*_ ≥ 30 events/h) is illustrated in Figure 2. For those with technically acceptable data (*n* = 99), the median evaluation period was 406 (298–462) min. Group median (IQR) values were: (1) AHI_AL_: 10.9 (4.9–22.2) events/h, (2) ODI_AL_: 14.0 (6.6–23.8) events/h, and (3) Tsat_AL_ < 90%: 7.9 (1.4–27.9%) events/h.

### 3.5 Screening performance metrics

#### 3.5.1 SBQ scores

*An SBQ*≥3 au, produced modest PPVs for predicting both *moderate‐to‐severe* and *severe OSA*, *but* a high NPV for severe OSA (see Table 3).

**TABLE 3:** Positive (PPV) and negative (NPV) predictive values for predicting moderate‐to‐severe and severe OSA when using an SBQ score ≥3 au only (*n* = 137), AHI_AL_ thresholds = 10, 15 or 30 events/h (*n* = 99), or a combination of SBQ score ≥3 au and AHI_AL_ thresholds = 10, 15 or 30 events/h (*n* = 53)

	AHI_PSG_ ≥ 15		AHI_PSG_ ≥ 30	
	PPV	NPV	PPV	NPV
SBQ only				
SBQ ≥3 au	63.4	69.1	39.0	92.7
AL only				
AHI_AL_ = 10	61.1	75.6	33.3	95.6
AHI_AL_ = 15	65.8	68.9	44.7	95.1
AHI_AL_ = 30	72.7	59.1	63.6	85.2
SBQ & AL				
AHI_AL_ = 10	60.6	50.0	42.4	90.0
AHI_AL_ = 15	69.6	53.3	56.5	90.0
AHI_AL_ = 30	80.0	48.8	70.0	79.1

*Note*: Values expressed as percentages (%).

Abbreviations: AHI, Apneoa‐hypopnea index; AHI_AL_, AHI from ApneaLink; AHI_PSG_, AHI from polysomnography; AHI_PSG_ ≥ 15 events/h, moderate‐to‐severe OSA; AHI_PSG_ ≥ 30 events/h, severe OSA; AL, ApneaLink; NPV, negative predictive value; PPV, positive predictive value; PSG, polysomnography; SBQ, STOP‐Bang questionnaire.

#### 3.5.2 ApneaLink™Air data

Across all threshold levels, PPV values were at the most modest for predicting both moderate‐to‐severe and severe OSA. NPVs were highest for predicting severe OSA, but lower for predicting moderate‐to‐severe OSA (Table 3).

#### 3.5.3 Combination of SBQ and ApneaLink™Air data

The combination of the SBQ ≥ 3 au threshold and AHI_AL_ thresholds of 10, 15 or 30 events/h did not substantially improve NPV or PPV values when compared with those achieved for each tool alone. There was only a slight improvement in PPV for predicting moderate‐to‐severe and severe OSA (Table 3).

#### 3.5.4 Cancer type and patient age

**FIGURE 3:**
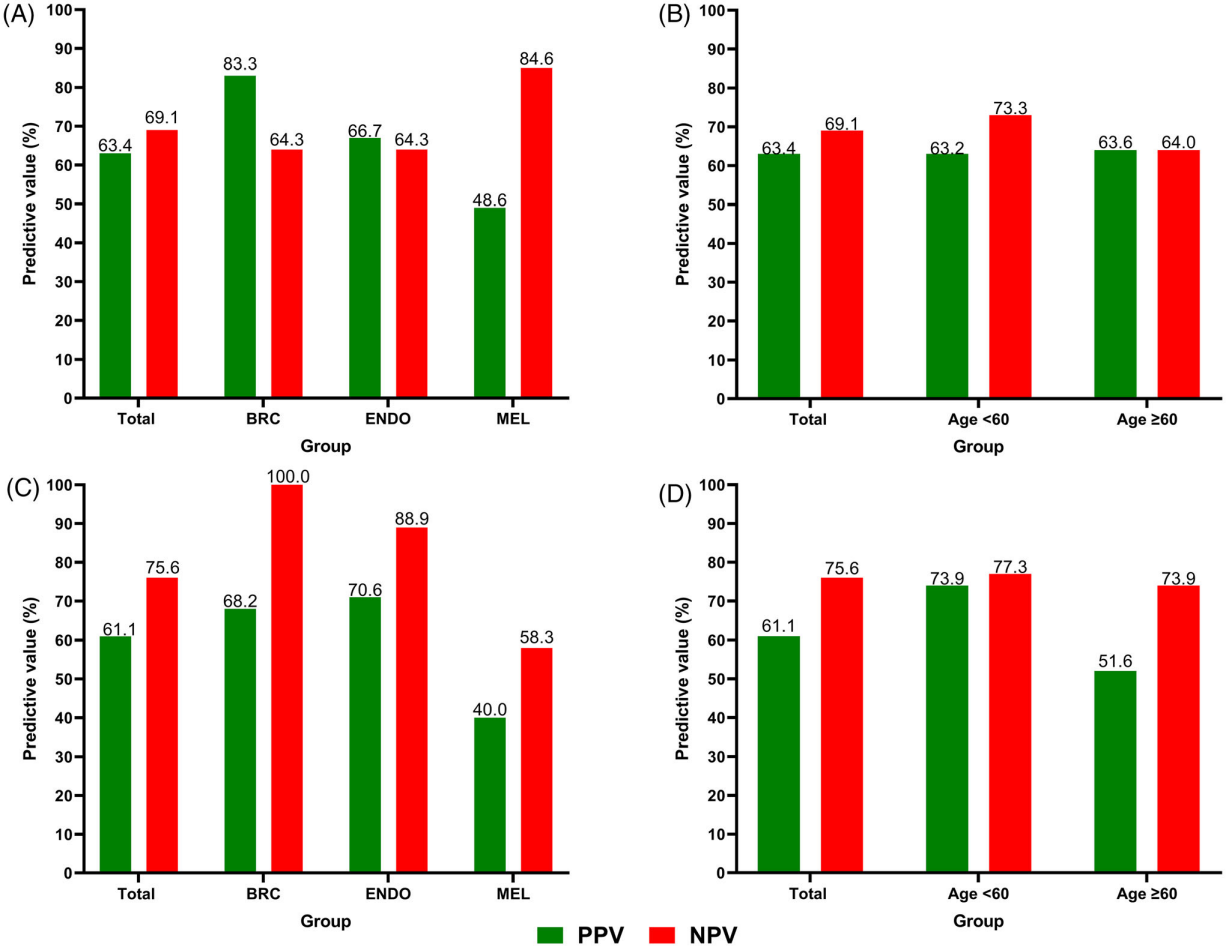
Bar plots of positive predictive (PPV; red) and negative predictive values (NPV; green) for moderate‐to‐severe OSA (AHI_PSG_ ≥ 15 events/h) when using a STOP‐Bang (SBQ) score ≥3 au across (A) cancer sub‐groups (*n* = 137; BRC, *n* = 52; ENDO, *n* = 35; MEL, *n* = 50), and (B) age sub‐groups (*n* = 137; Age < 60, *n* = 68; Age ≥ 60, *n* = 69); and for an ApneaLink™*Air* AHI threshold ≥10 events/h (AHI_AL_ ≥ 10 events/h) across (C) cancer sub‐groups (*n* = 99; BRC, *n* = 34; ENDO, *n* = 26; MEL, *n* = 39), and (D) age sub‐groups (*n* = 99; Age < 60, *n* = 45; Age ≥ 60, *n* = 54). BRC, breast cancer; ENDO, endometrial cancer; MEL, melanoma

Figure 3 displays PPV and NPV for SBQ ≥ 3 au and AHI_AL_ ≥ 10 events/h for predicting an AHI_PSG_ ≥ 15 events/h, by cancer type and patient age. The lowest PPV values, for both SBQ ≥ 3 au and AHI_AL_ ≥ 10 events/h, occurred in the melanoma and the age ≥ 60 years sub‐groups.

## 4 DISCUSSION

Cancer patients often complain of poor sleep and sleep‐related symptoms (e.g., difficulty falling asleep, daytime sleepiness, unrefreshing sleep, snoring, fatigue).^42^ We recently published data describing four distinct sleep symptom clusters identified across 318 cancer patients; a minimally symptomatic group (47.7%); insomnia‐predominant group (24.9%); very sleepy with upper airway symptoms (16.3%), and a severely symptomatic group with severe dysfunction (11.1%).^43^ Breast cancer patients were more likely to report insomnia related or severe symptoms, whereas melanoma patients were more likely to be minimally symptomatic or sleepy with upper airway symptoms. Endometrial cancer patients were equally distributed across symptom clusters. These sleep symptom clusters overlap with similar symptom clusters reported for OSA patients.^44^ Consequently, there is a need to identify whether cancer patient sleep symptomology is related to co‐morbid OSA and is, therefore, specifically treatable. OSA screening may provide a tool for oncologists to identify which patients warrant referral to a sleep physician for evaluation.

The purpose of the present methodological study was to validate SBQ and ApneaLink™*Air* for use as OSA screening methodologies when applied in an oncology clinic setting. There are no previous publications that report SBQ and ApneaLink™*Air* performance data for cancer clinic cohorts. All previous validation studies for these tools were conducted in sleep/surgical clinics or community settings.^41^, ^45^, ^46^, ^47^, ^48^, ^49^, ^50^, ^51^, ^53^, ^54^, ^55^, ^56^

The present study demonstrated that, in cancer patients, either SBQ or ApneaLink™*Air* were modestly effective at excluding patients without severe OSA, with no additional benefit gained when using them in combination. However, both methods were ineffective at identifying patients with moderate‐to‐severe or severe OSA. The prevalence of moderate‐to‐severe OSA in this older, obese, predominantly female cohort (*n* = 143) was nearly 50% (71 patients), with 37 patients having severe OSA. This prevalence falls at the higher end of the range reported for the general adult (21.2%–49.7%),^18^, ^19^ and is higher than for the general female population (13.2%–23.4%).^18^, ^19^ Indeeed, prevalence rates arising from PSG data in the present study, approach those reported in sleep‐clinic cohorts,^20^, ^21^, ^22^, ^23^ suggesting OSA prevalence in cancer patients may be as high as in the high‐pretest probability environment of a sleep physician referral clinic. Furthermore, given that OSA was not previously clinically recognised in any of the cancer patients included in the present study, these findings emphasise the nature of the unmet need for OSA screening in cancer patients.

### 4.1 STOP‐Bang questionnaire

The SBQ is a straightforward, easy‐to‐administer OSA screening tool with a simple scoring system.^33^ The tool has been validated across many clinical settings^33^, ^57^ and adopted in certain clinical guidelines^32^ for the appropriate triage of patients with suspected OSA for further clinical assessment. The majority (*n* = 82; ~60%) of patients in the present study who completed the SBQ (*n* = 137) scored ≥3 au, indicating most patients had an elevated OSA risk.^49^ SBQ≥3 au had good discriminative power (NPV ≈ 93%) to identify patients without severe OSA, but performed poorly for positively identifying those with severe OSA (PPV = 39%). This trend of high NPV values and low PPV values, resembles previously reported data for sleep clinic,^52^ surgical clinic,^34^ and general population‐based cohorts.^46^

A low PPV for SBQ and may be related to: (1) underlying prevalence of OSA in the particular study cohort,^34^ or the (2) gender‐specific technical issues with STOP‐Bang.^50^, ^54^ The present cohort contained a high proportion of female participants who can only obtain a maximum SBQ score of 7 au. SBQ has been previously demonstrated to underestimate the risk for OSA in female populations.^50^, ^54^ When matched for OSA severity, males tend to produce a significantly higher mean SBQ score than females, a finding attributed to the SBQ being focused on the male gender and typical male OSA symptoms.^57^ However, despite this SBQ performed similarly in our predominantly‐female, oncology clinic‐recruited cohort to previous reports^46^, ^51^ assessing mixed sex cohorts recruited from other clinical settings. Consistent with previous reports, overall, an SBQ≥3 au. cutoff in the present study effectively identified patients unlikely to have severe OSA.^51^

### 4.2 ApneaLink™Air

The failure rate for obtaining technically acceptable ApneaLink™*Air* data from those who received the device (*n* = 117) was 15.4%, this includes patients who were provided the device but did not subsequently use it. For those who used the device (*n* = 105), the technical failure rate was 5.7%, similar to previous reports for cohorts recruited from sleep clinics or other settings.^23^, ^47^, ^52^, ^58^

Based on ApneaLink™*Air* data alone, moderate‐to‐severe OSA was present in ~38% of patients (*n* = 38) and severe OSA in ~11% (*n* = 11). These prevalence rates are less that obtained via PSG, for the same patient cohort. ApneaLink™*Air* had moderate utility in predicting moderate‐to‐severe OSA, but greater utility for identifying patients without severe OSA, particularly when using an AHI_AL_ ≥ 10 events/h threshold. The PPV for predicting an AHI_PSG_ ≥ 15 events/h using the AHI_AL_ ≥ 15 events/h threshold, in this study is similar to some previous validation studies,^55^ but not others.^48^, ^56^ The NPV for ApneaLink™*Air* for moderate‐to‐severe OSA when using the AHI_AL_ ≥ 15 threshold, was substantially lower than most previously reported values (68.9% vs. 51.4%,^56^ 82.5%^48^ and 93.5%^55^). The NPV for an AHI_AL_ ≥ 30 events/h threshold to identify patients without severe OSA was better than reported by Delesie et al., 2021^56^ (63.6% vs 70%^56^).

Differences between our study and with other validation studies that have used different ApneaLink™ devices may be a consequence of: (1) the prevalence of moderate‐to‐severe or severe OSA within the particular study cohort^50^; (2) the criteria used to score OSA‐related events,^40^, ^59^ (3) the minimum acceptable evaluation period,^53^ (4) the use of manual or automatic scoring methods,^47^ and (5) the particular version of the ApneaLink™ device used.^47^

### 4.3 SBQ and ApneaLink™Air data

A 2‐step screening model, developed to identify patients with moderate‐to‐severe OSA in a primary care setting using a questionnaire and the ApneaLink™*Air* device,^36^ has been recommended to streamline the utilisation of finite PSG resources.^32^ However, in the present study, combining SBQ and ApneaLink™*Air* data made little difference to screening outcomes (Table 3).

### 4.4 Patient age and cancer type

Both SBQ and ApneaLink™*Air* tools were both less effective at positively identifying those with moderate‐to‐severe OSA in the melanoma sub‐group (PPV for predicting AHI_PSG_ ≥ 15 events/h was 48.6% and 40.0% for SBQ≥3 au. and AHI_AL_ ≥ 10 events/h, respectively), with ApneaLink™*Air* being also less effective in the older age group (Figure 3). The reason for these outcomes is not clear from our data set.

### 4.5 Strengths and limitations

This is the first study to document performance characteristics for OSA screening tools, SBQ and ApneaLink™*Air* device, in an oncology clinic setting. Major strengths include the use of the ‘gold standard PSG’ to confirm OSA status and establish the ‘true’ prevalence of OSA in the study cohort, and experienced technician review of ApneaLink™*Air* data recording quality. Patients were recruited while attending for post‐treatment follow‐up, and ApneaLink™*Air* studies were performed unattended at home. Thus, this study reflects the ‘real world’ experience of deploying OSA screening tool(s) in clinical oncology settings.

Limitations include a small sample size, a restricted number of cancer types, and potential recruitment bias associated with volunteers self‐selecting for OSA symptoms thus potentially inflating the underlying OSA prevalence in the study cohort. Single‐night studies for PSG and ApneaLink™*Air* leave our dataset vulnerable to night‐to‐night variability;^59^, ^60^ however, single night PSG studies are the accepted clinical standard, making our study generalisable to actual clinical practice.^32^ Since our study featured two female‐associated cancers, our study cohort was predominantly female. Consequently, our findings might not be generalisable to other cancer types or to male‐dominant cancer cohorts.

## 5 CONCLUSION

In a cohort of breast, endometrial and melanoma cancer patients, SBQ data effectively identified patients unlikely to have severe OSA, and shared similar performance characteristics to ApneaLink™*Air* data. Both tools were less effective at positively identifying patients with moderate‐to‐severe or severe OSA. There was no improvement with a 2‐step combined tool approach. We conclude that PSG remains the optimal tool for the positive diagnosis and management of comorbid OSA in cancer patient cohorts.

## AUTHOR CONTRIBUTIONS

**Harini Subramanian:** Data curation (equal); formal analysis (lead); visualization (lead); writing – original draft (equal); writing – review and editing (lead). **Veronika Fuchsova:** Data curation (equal); formal analysis (lead); investigation (lead). **Elisabeth Elder:** Funding acquisition (supporting); resources (equal); writing – review and editing (supporting). **Alison Brand:** Funding acquisition (supporting); resources (equal); writing – review and editing (supporting). **Julie Howle:** Funding acquisition (supporting); resources (equal); writing – review and editing (supporting). **Anna deFazio:** Funding acquisition (supporting); resources (equal); writing – review and editing (supporting). **Graham J Mann:** Funding acquisition (supporting); resources (equal); writing – review and editing (supporting). **Terence Amis:** Conceptualization (equal); formal analysis (supporting); funding acquisition (lead); methodology (equal); project administration (equal); supervision (equal); visualization (supporting); writing – original draft (equal); writing – review and editing (lead). **Kristina Kairaitis:** Conceptualization (equal); formal analysis (supporting); funding acquisition (lead); methodology (equal); project administration (lead); supervision (equal); visualization (supporting); writing – original draft (equal); writing – review and editing (lead).

## CONFLICT OF INTEREST

The authors explicitly state that there are no conflicts of interest in connection with this article. Anna DeFazio declares receiving research grants and honoraria from AstraZeneca, although this is not in relation to the subject matter or materials discussed in this manuscript.

## ETHICS STATEMENT

All study procedures were approved by the Western Sydney Local Health District Ethics Committee, and were undertaken, adhering to the Declaration of Helsinki.

## Data Availability

All data collected in this study is primary data. Data will be made available, on acceptance of manuscript for publication.
